# Medical Association of the Southwest

**Published:** 1910-12

**Authors:** 


### Society.

### Medical Association of the Southwest.

The Medical Association of the Southwest met in parlor D, Hotel Eaton, Wichita, Kansas, October 11-12, 1910.

Meeting of the Executive Committee of the Medical Association of the Southwest at 8 a. m. with the following members present: Drs. G. H. Moody, J. F. Gzell, G. W. Maser, Howard Hill, D. A. Myers, S. G. Burnett, C. W. Fassett and F. H. Clark.

Dr. C. E. Bowers being unable to be present, Dr. J. F. Gzell made a statement of the plans for the meeting, outlining the program as already printed. He also reported that Rev. W. S. Priest was absent from the city and would not be able to be present.

The Secretary made an informal report of the work done in preparation for the meeting.

Meeting adjourned to meet October 12 at 8 a. m.

F. H. Clark, Secretary.

### GENERAL SESSION.

The fifth annual meeting of the Medical Association of the Southwest was called to order at 9 a. m. in the Scottish Rites Temple by Dr. Chas. E. Bowers, Chairman of the Committee of Arrangements. After the invocation, Dr. Bowers introduced Hon. C. L. Davidson, mayor of the city of Wichita, who welcomed those present on behalf of the citizens of Wichita. Dr. O. P. Davis, President of the Kansas State Medical Association, then welcomed those present in behalf of the State Association, and Dr. F. E. Oldham, on behalf of the local profession, after which Dr. Bowers introduced Dr. G. H. Moody, who in turn called upon Dr. Joe Becton of Texas to respond to the word of welcome, after which Dr. Bowers, as Chairman of the Committee of Arrangements, reported upon the program with especial reference to the social features which were to take place.

The President then appointed as a Committee on Resolutions, Drs. Jabez N. Jackson, E. H. Martin and W. L. Allison.

Dr. Jabez N. Jackson then moved, which was duly seconded and carried, that as many of the physicians had not yet arrived, we spend the remainder of the morning session in general scientific session, each Chairman presiding in turn.

Dr. H. M. Lyle, Chairman of the Section on General Medicine, called upon Dr. E. H. Thraikill, of Kansas City, who read a paper upon "Colica Mucose" with report of cases. This paper was freely discussed by Drs. Hertzer and Robinson, of Kansas City, and the discussion ended by the essayists.

In absence of Dr. Wm. Keiller, Chairman of the Section of Surgery, Dr. D. A. Myers, as Vice-Chairman, called upon Dr. D. W. Basham, who read a paper upon "The Post-operative Care and Treatment of Suprapubic Prostatectomy, with Especial Reference to the Method of Drainage and the Treatment of Shock and Hemorrhage." This was discussed by Drs. Jackson, Bransford Lewis, Mark, Griffeth, Becton, Hill and Norbery, and closed by the essayists.

Meeting adjourned until 2 p. m.

F. H. Clark, Secretary.

At the caucus of the various State delegations the following were nominated as members of the Nominating Committee:

From Texas—Drs. Joe Becton, J. E. Gilcreest, W. L. Allison and W. A. Wood.

From Arkansas—Drs. St. Cloud Cooper, E. H. Martin, J. M. Griffin and C. H. Cargile.

From Oklahoma—Drs. C. Thompson, E. S. Lain, J. H. Scott, D. A. Myers and J. H. Barnes.

From Missouri—Drs. G. W. Robinson, E. H. Thrailkill, Jno. Punton, A. W. McAlester and Bransford Lewis.

From Kansas—Drs. C. C. Goddard, J. W. May, S. E. Sawtelle, E. E. Liggett and R. Claude Young.

### Tuesday Evening, October 11, 1910.

General session called to order after a delightful informal musical and scenic program, furnished by the Committee of Arrangements, after which Dr. D. A. Myers, Vice-President, introduced Dr. G. H. Moody, who delivered the annual President's address, which was very timely, he using as his subject "Fatigue."

Dr. Edward H. Ochsner was then introduced and delivered a masterly address upon the "Prevention and Treatment of Septic Infection of the Extremities."

Among the many social programs provided by the physicians of Wichita, especial mention should be made of the program of musical numbers and the beautiful scenery of the Scottish Rites Temple. All enjoyed very greatly the splendid voice and musical numbers rendered by Mrs. Campbell, as well as the scenic effects produced through the kindness of the superintendent of the Temple.

Of course no one missed the banquet tendered the physicians and their wives and which took place immediately after Dr. Ochsner closed his oration.

The speakers for the evening were:

Dr. J. M. Griffin of Sulphur Springs, Ark., whose subject was "The Arkansas Traveler."

Dr. A. K. West of Oklahoma City, Okla., who told all about "The Sooner."

Dr. Bransford Lewis of St. Louis, Mo., who "Show'd Me."

Dr. S. S. Glasscoch of Kansas City, Kan., on "The Sunflower," and last, but not least,

Dr. Joe Becton of Greenville, Texas, who handled "The Lone Star" in his usual "Becton manner."

All joined in saying that the profession of Wichita had "done themselves proud" and had provided an evening's entertainment long to be remembered.

F. H. Clark, Secretary.

WEDNESDAY MORNING, OCTOBER 12, 1910.

Meeting of the Executive Committee with the following members present: Drs. G. H. Moody, H. Hill, J. H. Johnson, J. F. Gzell, J. H. Riddle, C. W. Fassett, S. G. Burnett and F. H. Clark.

A letter from Dr. B. J. Vane expressing his regrets at not being able to present was read and ordered placed on file.

The following resolutions were read by the Secretary:

WHEREAS, The National Pure Food law is in danger of failing in its purpose by technical interpretations which prohibits chemically preserved food products to be labeled as pure under the law; be it

*Resolved,* That the Medical Association of the Southwest condemns the use of antiseptic drugs, such as benzoate of soda and similar chemicals in food products designed for human consumption; and be it further

*Resolved,* That this Association heartily endorses the plan of a National Department of Public Health as a public necessity and urges its enactment by the coming Congress.

Upon motion, duly seconded and carried, the above resolutions were recommended to the general session and their adoption recommended.

The Secretary-Treasurer for the year just closed, I have only to report that in compliance with the instruction of the Executive Committee. At its last meeting I made due effort to publish the proceedings of the last annual session held in San Antonio, including the papers and discussions in pamphlet form, but I found that impracticable owing to the fact that I have not, as yet, received the stenographic report owing, I am informed, to the fact that the stenographer employed at the meeting was ill for some time following the meeting. I found also that the funds on hand would not warrant such an expense.

Acting through the Publication Committee, the papers were divided among the various State and private journals and they have all been published, I think.

I believe that the proceedings and papers and discussions should all be published in pamphlet form if sufficient funds can be secured for this purpose and a copy sent to every member whose dues have been paid for the present year.

The Western Surgical and Gynecological Society follow this plan with great satisfaction. Besides this the papers and discussions should be divided among the various State journals and in this way the members of each State association will have an opportunity to read them.

Some little criticisms having been made because of the sending out of statements for dues, no statements were sent out this year.

The financial statement shows about enough money on hand to pay all bills to date.

Following the custom of the past a circular letter, application for membership and a copy of the preliminary program were mailed to every member of the five State associations and as there are nearly 9000 of them it made quite a laborious task, I do not see how the Association can be kept before the men whom we hope to reach and their interest increased without this being done, as a personal letter seems to do more good than anything else, and as a result this year quite a number were received of new members.

The following made application for membership since our last meeting:

Dr. J. R. McCluggage, Augusta, Kan.; Dr. Harry Reding, Lawrence, Kan.; Dr. Leon Hatassarrin, Lawrence, Kan.; Dr. E. B. Dunlap, Lawton, Okla.; Dr. L. A. Jacobus, Winfield, Kan.; Dr. John H. Henson, Mound Valley, Kan.; Dr. L. J. Moorman, Oklahoma City, Okla.; Dr. Frank C. Neff, Kansas City, Mo.; Dr. R. Claude Young, Arkansas City, Kan.; Dr. C. F. Bucklin, Sawyer, Kan.; Dr. B. F. Hawk, Harper, Kan.; Dr. Francis I. Dodge, Ashland, Kan.; Dr. R. M. Howard, Oklahoma City, Okla.; Dr. C. S. Rannels, Cedar Point, Kan.; Dr. H. L. Scales, Hutchinson, Kan.; Dr. M. M. Roland, Oklahoma City, Okla.; Dr. T. H. Jamison, Wellington, Kan.; Dr. A. H. Willard, Cyril, Okla.; Dr. Wm. T.

Grove, Eureka, Kan.; Dr. W. Eugene Dixon, Oklahoma City, Okla.; Dr. F. S. Williams, Wichita, Kan.; Dr. J. M. Cooper, Enid, Okla.; Dr. F. K. Slaton, Goltry, Okla.; Dr. W. H. Graver, Wichita, Kan.; Dr. Fred W. Duncan, Coffeyville, Okla.; Dr. C. D. Fooney, Wichita, Kan.; Dr. J. W. Risdon, Leavenworth, Kan.; Dr. C. W. Gisney, Kansas City, Mo.; Dr. A. S. Risser, Blackwell, Okla.; Dr. A. P. Gearheart, Blackwell, Okla.; Dr. H. S. Hickok, Wichita, Kan.; Dr. A. E. Milker, Shawnee, Okla.; Dr. F. M. McCallum, Kansas City, Mo.; Dr. G. M. Wooden, Anthony, Kan.; Dr. Z. T. Clark, Cherokee, Okla.; Dr. W. T. Jank, Chautauqua, Kan.; Dr. A. H. Horan, Wichita, Kan.; Dr. Geo. R. Little, Wichita, Kan.; Dr. H. G. Welch, Hutchinson, Kan.; Dr. W. R. Teverton, Cloud Chief, Okla.; Dr. R. G. Nelson, Elm Ridge, Kan.; Dr. S. T. Shelly, Mulvane, Kan.; Dr. J. Z. Hoffman, Wichita, Kan.; Dr. A. H. Nossaman, Wichita, Kan.; Dr. S. A. Nossaman, Cunningham, Kan.; Dr. J. H. Staatz, Burghton, Kan.; Dr. M. Trueheart, Sterling, Kan.; Dr. E. M. Seydell, Wichita, Kan.; Dr. W. H. Addington, Altoona, Kan.; Dr. J. M. McGuire, Noodsha, Kan.

The above are all members of their respective State associations and are therefore eligible for membership.

| | |
|---|---|
| Balance on hand at last report | $122 84 |
| Received from dues | 408 00 |
| Total | $530 84 |
| Expended for printing bill | $ 88 50 |
| Incidentals | 12 40 |
| Clerical help | 117 00 |
| Postage | 123 73 |
| Balance on hand | 189 21 |
| | $530 84 |
| Unpaid bills, printing | $100 00 |
| Wm. Whitford | 94 55 |

Respectfully submitted,

F. H. Clark, Secretary.

On motion, duly seconded and carried, the report was accepted and ordered embodied in the report of the Executive Committee to the general session.

The question of publishing the proceedings was discussed at length by all present and a proposition for publishing the same was made by Dr. C. W. Fassett, which was fully discussed by Dr. Moody, Riddle, Burnett, Clark, Gzell, Fassett and Hill.

Dr. Gzell moved that the Secretary publish the proceedings of this session in pamphlet form, which motion was duly seconded and carried.

On motion the meeting adjourned.

WEDNESDAY EVENING, OCTOBER 12, 1910.

Meeting called to order by President G. H. Moody. The Secretary gave the report of the Executive Committee recommending:

1. The adoption of the resolutions asking Congress to pass an act to stop the use of antiseptic to preserve food and create a Department of Public Health.

2. To publish the proceedings in pamphlet form.

3. Accepting the report of the Secretary-Treasury as read. On motion, duly seconded and carried, the report was accepted and the recommendations adopted.

Moved by Dr. Jackson and duly seconded and carried, that the Chairman of the Committee of Arrangements for the future meetings be requested to eliminate all addresses of welcome and responses from the program.

Moved by Dr. Jackson, duly seconded and carried, that at the next annual meeting the Committee provide one General Scientific Section.

The following resolutions were read:

We your Committee appointed to draft resolutions beg leave to submit the following:

*Resolved*, That as an evidence of our appreciation, the thanks of this Association be extended to the Kansas State Medical Association, the Eastern Star, the Scottish Rites bodies, Mrs. Jetta Campbell Stanley and the Midian Quartette for the magnificent and generous entertainment extended to us at this our fifth annual meeting, and particularly do we express our appreciation to Dr. Bowers and his associates, through whom these courtesies have been obtained. The city of Wichita has proven itself progressive beyond our most sanguine expectations and the genial hospitality of its citizens will ever remain as a bright memory in the minds and affections of each of us who has had the good fortune to be here.

JABEZ N. JACKSON,
E. H. MARTIN,
W. L. ALLISON,
Committee.

On motion the report of the Resolutions Committee was unanimously adopted and ordered spread upon the minutes.

The Nomination Committee reported as follows for offices for the coming year:

President—Dr. M. L. Perry, Parsons, Kan.

Vice-President—Dr. J. M. Griffin, Sulphur Springs, Kan.

Vice-President—Dr. W. H. Stauffer, St. Louis, Mo.

Vice-President—Dr. W. L. Allison, Fort Worth, Texas.

Secretary-Treasurer—Dr. F. H. Clark, El Reno, Okla.

For members of the Executive Committee to serve three years:

Dr. W. A. Wood, Hubbard, Texas; Dr. S. S. Glasscoch, Kansas City, Kan.; Dr. J. H. Scott, Shawnee, Okla.; Dr. St. Cloud Cooper, Fort Smith, Ark.; Dr. J. D. Griffeth, Kansas City, Mo.

For place of meeting, 1911, Oklahoma City, Okla.

On motion, duly seconded and carried, the report was adopted and the officers declared duly elected.

The President then appointed Drs. Jackson and Becton a committee to conduct the President-elect to the chair. In a few well chosen words Dr. Perry thanked the members for the honor they had conferred upon him.

Dr. S. S. Burnett then gave notice that at the next annual meeting he would move to amend the by-laws so that the Publication Committee shall be instructed to divide the papers between the various medical journals of this organization.

On motion the meeting adjourned.

F. H. Clark, Secretary.

### Section Officers, 1911.

Surgery—Chairman, Dr. J. F. Kuhn, Oklahoma City, Okla.; Vice-Chairman, Dr. Spitler, Wellington, Kan.; Secretary, Dr. Howard Hill, Kansas City, Mo.

General Medicine—Chairman, Dr. C. C. Conover, Kansas City, Mo.; Vice-Chairman, Dr. A. D. Young, Oklahoma City, Okla.; Secretary, Dr. G. W. Robinson, Kansas City, Mo.

Eye, Ear, Nose and Throat—Chairman, Dr. H. C. Todd, Oklahoma City, Okla.; Vice-Chairman, Dr. J. H. Barnes, Enid, Okla.; Secretary, Dr. J. W. May, Kansas City, Kan.

### Meeting of the Executive Committee, October 12, 1910.

The following members were present: Drs. M. L. Perry, President; E. H. Martin, S. G. Burnett, C. W. Fassett, E. S. Lain, S. S. Glasscoch, W. A. Wood, J. F. Gzell and F. H. Clark.

The Committee instructed the Secretary in preparing the program for the coming year to arrange for a two and one-half day session. The first evening session to be devoted entirely to the scientific program and the second evening to social features.

The President appointed as a Publishing Committee for the coming year:

Dr. Claude Thompson, Muskogee, Okla.; Dr. Morgan Smith, Little Rock, Ark.; Dr. J. W. May, Kansas City, Kan.; Dr. Holman Taylor, Fort Worth, Texas; Dr. E. J. Goodwin, St. Louis, Mo.

On motion meeting adjourned.

F. H. Clark, Secretary.

Reported for the "Red Back."

